# 

**Published:** 1868-01

**Authors:** 


					﻿Rental Btjista
OF THE WEST,
Issued Monthly, at $3 a Year in Advance.
DEVOTED TO THE INTERESTS OF THE DENTAL PROFESSION.
EDITED BY J. TAFT AND G, WATT,
The volume commences in January and closes in December.
The Register being issued monthly is always fresh, therefore indispensable to the practi-
tioner who5desires to keep posted up with the new inventions and improvements which are
daily developed. Neither expense nor effort will be spared to give value and variety to its
contents. Articles will be illustrated with well executed engravings whenever they will add
to the interest or assist in explanation.
Its extensive circulation and frequency of issue makes it one of the BEST DENTAL
ADVERTISING MEDIUMS in the United States.
RATES OF ADVERTISING.
One page, lyear, $50. Half page, 1 year, $30. Quarter page, or card, 1 year $20.
All advertising bills payable in advance, or at furthest after the issue of the first number
containing the advertisement. All transient advertisements to be paid for in advance.
esr- CONTRIBUTIONS SOLICITED.
Address, J. TAFT, or “DENTAL REGISTER," Cincinnati Ohio.
Business Communications to
TAFT, Publisher*,
No. 238 Fourth St., between Plum and Central Avenue
NEW VOLUME.
With this number commences the 22d volume of the Dental
Register, and with it we cheerfully send forth to its many
readers our New Year’s greeting, trusting that in all respects
they may have a happy and prosperous year; and especially
may they be blessed in that which they may be able to gather
from the pages of the Register. We entertain a goodly hope
that to most of the readers it has been not only a welcome
visitor, but a profitable messenger. None know better than we,
its faults and shortcomings ; but we shall use every possible effort
to rejnedy the defects, and make it better in every respect.
We desire that it shall be a faithful representative of the van-
guard of the profession. Whatever our hopes and aspirations
may be, its character and efficiency for good will be greatly
modified by those who contribute to its pages.
We shall hope for the largest share of patronage in this
respect for the coming volume.
There is too, another kind of patronage we can not afford to
give up,—pecuniary. Our readers will bear in mind, that the
design is advance payment; necessity almost compels this, and
the subscription can just as easily be paid at the beginning, as
at the end of the volume ; which would be a very great accom-
modation to the publisher. A few are in arrears for two or three
years; the names of all such will be cut off unless we hear from
them before another issue. We sent bills with the December
number to all in arrears to that time, and with this number send
bills to all still delinquent, and also for the present volume, to
which we trust a hearty response will be made at once.
The more promptly the subscriptions are paid, the better will
be the Journal, and the more profitable to the reader.
We trust these few lines will be read very attentively by all
the subscribers.	T.
MISSISSIPPI VALLEY DENTAL ASSOCIATION.
Tiie annual convocation of this the oldest Dental society in
existence, will be held in the lecture room of the Ohio Dental
College, beginning its sessions on Wednesday, March 4th, at
10 o’clock, a. M., when we shall hope to greet all those who have
been accustomed for so many years, to come up to these gather-
ings; and we most earnestly invite all to come. Only those who
have been accustomed to be present know how good it is to be
there, and those who have never been present at these meetings,
can only know how profitable they are by being there. Then let
all come.
The Secretary’s notice will be found on another page. T.
OHIO DENTAL COLLEGE ASSOCIATION.
The annual meeting of this body will be held on Monday the
2d of March, at 2 o’clock, p. m., when it is hoped every member
of the association as far as possible will be present.
This association having in charge the general management and
guidance of the college, should have as full a representation of
its membership as possible. The institution is in one sense in a
transition state, passing on to a much higher position in its
method of operation than it has heretofore attempted, and it
needs the wisdom and co-operation of all its friends, and those
to whom it belongs. Without the sympathy and substantial
assistance of those who profess to be its lriends, it can hardly be
expected to maintain the advance it has already made ; but let
every member of this association do his duty, and a glorious
future is before the institution.
Let this be the largest meeting of the stockholders that has
ever been held.
The meetings will occupy Monday afternoon and evening, and
Tuesday to adjourn probably on Tuesday evening.	? T.
CLOSE OF THE COLLEGE SESSION.
The commencement exercises of the Ohio College of Dental
Surgery, will be held in the lecture room of the college, on Wed-
nesday evening, March 4th, at 7^ o’clock, at which time all the
friends of the institution and the public are invited.	T.
				

